# The Growing Phenomenon of ‘Frozen’ Virus Genome Sequences and Their Likely Origin in Research Facility Escapes

**DOI:** 10.3390/microorganisms12122412

**Published:** 2024-11-24

**Authors:** Steven E. Massey

**Affiliations:** Biology Department, University of Puerto Rico-Rio Piedras, San Juan 00931, Puerto Rico; steven.massey@upr.edu

**Keywords:** H1N1, polio, Ebola, Venezuelan equine encephalitis virus, EV71, frozen sequence, RNA virus

## Abstract

‘Frozen’ virus genome sequences are sampled from outbreaks and have unusually low sequence divergence when compared to genome sequences from historical strains. A growing number of ‘frozen’ virus genome sequences are being reported as virus genome sequencing becomes more common. Examples of ‘frozen’ sequences include the 1977 H1N1 ‘Russian’ flu; Venezuelan Equine Encephalitis Virus from Venezuela and Colombia in 1995; E71 sequences from a Hand, Foot and Mouth outbreak in 2007–2009 in China; and a polio strain isolated in 2014 from Anhui, China. The origin of these ‘frozen’ sequences has been attributed to escapes from research facilities and often appears to be associated with vaccine work. Consequently, a new paradigm for pathogen emergence appears in operation, that involves laboratory research or vaccine production which utilizes ‘live’ virus isolates of historical strains. The accidental release and re-emergence of such strains are straightforward to detect from their genome sequences and should spur the routine sequencing and publication of all known pathogenic viral strains undergoing experimentation, or being used for vaccine manufacture, in order to facilitate tracing. However, it is noted that novel pathogenic viruses accidentally released into the population from research facilities are harder to detect if their sequence has first not been made public, which should prompt the routine sequencing and reporting of all novel pathogenic viruses before experimentation.

## 1. Introduction

There are an increasing number of ‘frozen’ virus genome sequences being reported, which have been isolated during outbreaks, and which show high sequence identity to genome sequences of historical virus strains [1,2,3,4,5]. To date, all of these ‘frozen’ sequences have involved RNA viruses, which have high mutation rates [6]. The high mutation rate means that substantial sequence divergence from historical sequences would be expected if the virus was circulating in a host animal after the date of isolation of the historical strain. The existence of a ‘frozen’ genome sequence implies that the evolution of the virus has been arrested in some manner before re-emergence. RNA viruses do not persist long in the environment outside of a host [7,8], which implies that the ‘frozen’ virus genome has undergone some form of human-mediated storage. While an alternative possibility is that RNA viruses can undergo extended latency in a host animal, currently, there is no direct evidence to support this hypothesis for those virus species associated with ‘frozen’ genomes. In addition, given that some of the ‘frozen’ sequences match historical sequences from several decades prior, such an extended period of latency in a single animal is highly unlikely in these cases.

Here, all known ‘frozen’ viral sequences are described, and their connection to escapes from research facilities is explored. Suggestions are made as to how to encourage the use of routine genome sequencing in order to facilitate the tracing of accidental releases of viruses from scientific facilities to rectify biosafety weaknesses.

## 2. 1977 ‘ Russian’ H1N1 Flu

The first example of a ‘frozen’ virus sequence was that of the H1N1 influenza strain causing the 1977 ‘Russian’ flu pandemic. This was first reported to the World Health Organization (WHO) by the Soviet Union in December 1977, with the first person in Russia infected with the strain being identified on 1st November 1977 [9]. However, the virus had been earlier isolated in May/June 1977 in Liaoning Province and Tientsin Municipality, China [10]. It is notable that the H1N1 subtype went extinct in 1957 [11], which made its sudden re-emergence in 1977 remarkable. This was consistent with the observation that people younger than 20 were the most affected [12], indicating that they lacked immunity. Surprisingly, the genes of the H1N1 strain were found to be almost identical to those of H1N1 strains isolated in 1950 [1,13]. This indicated that the strain had been under storage until release, which sequence divergence indicated was about a year before it was first detected in 1977, which makes determining the exact geographical origin difficult [14]. Given the high level of sequence conservation with an H1N1 strain from 1950, the most likely origin of the 1977 H1N1 outbreak is a lab accident [11,14,15,16,17] or escape from a live-vaccine trial [15,18].

## 3. 1995 Venezuelan Equine Encephalitis Virus in Venezuela

The genome of the Venezuelan equine encephalitis virus (VEEV) subtype 1C strain, which caused a VEEV outbreak in 1995 in Venezuela and Eastern Colombia, showed a high degree of sequence identity (98%) to 1C strains from a 1964–1965 outbreak in the same region [2]. Only four nucleotide differences were noted between the earliest isolate from the 1995 outbreak (6119) and a strain from 1963 (P676-ag) [2]. P676-ag appears to be closely related to the progenitor of 6119. Incompletely inactivated subtype 1AB strains used for inactivated virus vaccines appear to have led to some previous VEEV outbreaks in Venezuela, given that almost total sequence stasis was observed between strains isolated from 1938 to 1973 [19]. However, subtype 1C strains were not used for the production of vaccines [2], which rules out this mode of entry into the horse population. ‘Live’ P676-ag was used for serology at the National Institute for Hygiene in Caracas [2], which implies that the source of the outbreak was a laboratory that utilized a ‘live’ P676-ag isolate in the region.

## 4. 2007–2009 Hand, Foot and Mouth Disease in Beijing, China

Fourteen enterovirus 71 (EV71) strains of Hand, Foot and Mouth Disease (HFMD) from an outbreak in China in 2007–2009 were found to have high sequence identity to the 1970 strain BrCr from the USA [3]. All fourteen strains and BrCr were found to have 97–100% nucleotide identity across the major capsid protein VP1 gene. Prior to the outbreak, BrCr was the sole representative of E71 genotype A; however, the outbreak represented an expansion of this clade. BrCr is the prototype strain of E71 [20], and BrCr isolates are commonly used in E71 research (for example see [21,22,23]). Consequently, it appears likely the outbreak in 2007–2009 stemmed from the release of BrCr or a close derivative from a research facility.

## 5. 2014 Polio in Anhui, China

In 2014, poliovirus was isolated by the Wuhan Institute of Virology from a fecal sample of a 14-year-old boy from Anhui province and was termed ‘WIV14’ [24]. Its genome sequence was subsequently shown to be 99% identical to that of the Saukett A strain [4], isolated from a boy in California in 1950 by Jonas Salk [25]. Saukett A is used in the production of the Inactivated Polio Vaccine (IPV) and is, as the name suggests, inactivated before incorporation into the vaccine. The striking level of identity between Saukett A and WIV14 indicates that Saukett A is the progenitor of WIV14. However, given that Saukett A was isolated over 60 years prior to WIV14, it implies that WIV14 had undergone an extended period of storage before release into the human population. As Saukett A is used for the production of IPV [26] and is readily available from virus biobanks [4], it is likely that it was released from a research facility or vaccine production center [4]. One scenario is that it was not fully inactivated before incorporation into an IPV.

The 1% sequence divergence is likely due to its circulation in the human population after release. An estimate was made of the synonymous substitution rate (Ks) in the P1/capsid of Saukett A (Accession PP972258) compared with WIV14 (Accession KY703697), using the method of [27] and the yn00 program of the PAML package [28]. Using this approach, Ks was calculated as 0.0645. A molecular clock Ks value of 0.032 per year for the poliovirus P1/capsid protein (nucleotides 1-2643) was calculated by [29] and indicates that the progenitor of WIV14 was released approximately 24 months prior to the date of sampling.

## 6. 2021 Guinea Ebola Outbreak

In 2021 an Ebola outbreak began in Gouéké, Guinea [30], 200 km away from the village of Guéckédou where the 2013–2016 West African Ebola outbreak apparently began [31] (although see [32]). Surprisingly, Ebola genome sequences from the 2021 outbreak were closely related to genome sequences from the 2013–2016 outbreak [5]. On a phylogenetic tree, the branch connecting genome sequences from 2021 is separated by only 12 nucleotide differences from genome sequences from 2014. The authors of the study note that there appears to be a new paradigm for transmission, suggesting that latency or dormancy of the virus in a host might be responsible for the retardation in genome sequence evolution. However, there is no evidence that Ebola can undergo multi-year latency in a human (the natural host remains to be determined [33]).

There are facilities that store Ebola samples in West Africa (such as the Kenema Government Hospital in Sierra Leone [34]), and the United States Agency for International Development (USAID) conducted extensive sampling from both humans and animals in the Forest Region of Guinea, where Gouéké is located [35]. The possibility that an Ebola-containing sample was the source of the outbreak remains to be explored. Of relevance to this hypothesis, the improper handling of Ebola samples was a cause of concern prior to the 2021 outbreak [36].

## 7. Are There Alternative Explanations for ‘Frozen’ Virus Sequences?

A ‘frozen’ virus genome sequence implies that the virus concerned has undergone a period of dormancy. Circulation in the human population, or in a reservoir species, would cause sequence divergence, as the virus would be actively replicating and accumulating mutations. The residence of the virus in a freezer is a possibility, but this is unlikely to occur outside a research facility. The long-term storage of RNA viruses is generally achieved via the use of low temperatures (−20 °C or −70 °C), but this generally requires a buffer containing protectants such as serum proteins [37]. While there is evidence for the long-term survival of DNA viruses in permafrost [38,39], DNA viruses tend to be more stable than RNA viruses. The examples of ‘frozen’ sequences described here exhibit an arrest in virus sequence evolution over a span of decades, and the permafrost scenario only seems applicable to the ‘Russian’ flu outbreak. However, while avian influenza appears stable in frozen water over a period of 1 year, which may reflect adaptation to a transmission conduit in Northern lakes [40], longer time periods would be expected to result in a drop in viability.

‘Biological stasis’ or viral latency in a host animal has also been mooted as a possible explanation behind retarded virus sequence evolution, such as in the case of the 2021 Guinea Ebola outbreak [5]. However, there is little supporting evidence for this conjecture, and the putative mechanism by which it might occur remains speculative. The persistent infection of animals by RNA viruses typically leads to rapid genetic sequence change rather than evolutionary retardation [41,42]. In the example of VEEV, the time span between the isolation of the historical strain and its re-emergence (29–30 years) is longer than the life span of its hosts: small mammals, horses and mosquitos [2]. This is inconsistent with latency in a single animal. Likewise, in the case of the 1977 ‘Russian’ flu, there were 27 years between the historical and re-emergent strains, which is hard to reconcile with a single host. In the example of poliovirus given here, the only natural host is human. Since the re-emergent strain was isolated from a 14-year-old boy in 2014, it would have needed at least one human–human transmission since its progenitor, Saukett A, was isolated in 1950, assuming that it had been circulating in the human population since that time. This implies that there should have been substantially greater sequence divergence over that time period than was observed.

## 8. What Action Should Be Taken When a ‘Frozen’ Virus Sequence Is Detected?

While there is a growing number of examples of ‘frozen’ sequences, all of which are listed here, these are likely the tip of the iceberg, given that virus genome sequences are only available for a subset of historical viral stocks. This suggests that more will be identified in the future with increases in virus genome sequencing. An immediate investigation to identify the originating facility should be conducted as soon as a ‘frozen’ sequence is reported. As can be observed from the listed examples, this problem is a global one. Sadly, given the inconvenience that conducting such an investigation entails, there might be negative consequences for those reporting the detection of ‘frozen’ sequences. A potential solution to this is to offer an international financial reward to or confer scientific recognition on those who identify and report such ‘frozen’ sequences.

## 9. Measures to Enhance Traceability of Escaped Viruses

One measure to enhance the traceability of putative escapes of viruses from research facilities would be to require the sequencing of all strains undergoing experimentation or involved in vaccine manufacturing. In both live attenuated virus vaccines and inactivated virus vaccines, the constituent virus must be amplified via passaging through cell lines or animal embryos before incorporation into the vaccine [43]. During passaging, RNA viruses rapidly pick up mutations [44], so these may represent unique identifiers that can be used to trace releases from vaccine facilities to a specific vaccine (after it has undergone amplification).

While the measures discussed above are applicable to known viral pathogens, a problem arises when a research facility experiments on novel viral pathogens, for which there are no sequence data available in a publicly accessible database. Of note, it has been argued that SARS-CoV-2 is unlikely to represent an escape from a research facility as it did not match any publicly available sequences [45]. However, a lack of access to a key viral sequence database [46] means that this claim is unverifiable.

Therefore, a solution is to require the publication of the genome sequences of novel virus isolates before beginning experimentation and the deposition of the sequences in a public tamper-proof (blockchain) database. Some novel viruses are not directly isolated but are only known from sequences in metagenomic datasets. These sequences can be used to produce a ‘live’ virus by inserting the genome sequence into an infectious clone construct. Such infectious clones could additionally be traced by the inclusion of a signature sequence into the virus genome sequence, such as leaving in restriction sites by avoiding the use of seamless cloning, or by adding a short sequence tag. Currently, these measures are not implemented for infectious clone constructs; consequently, ‘live’ viruses generated from infectious clones would be difficult to trace if they are accidentally released and if the virus genome sequence has not been previously reported.

## 10. Conclusions

The growing list of ‘frozen’ virus genome sequences indicates a new paradigm for the emergence of pathogenic viruses and the origin of viral disease outbreaks, namely via escape from research facilities. Such facilities may be dedicated solely to research or may be involved in vaccine manufacturing; however, a common factor is that they work with ‘live’ virus isolates of historical strains. As more viral genome sequences are generated, it is expected that more examples will be identified. The examples of ‘frozen’ virus genomes listed here indicate that lapses in virus lab biosafety are a worldwide problem. However, on a positive note, a mechanism is provided via the use of genomics to monitor lab biosafety and detect escapes when they occur. The key recommendations of this work are that genome sequences of ‘live’ isolates of known and novel virus strains should be made public before experimentation begins, that infectious clone constructs should be made traceable using sequence signatures and that the surveillance and detection of ‘frozen’ virus genome sequences should be incentivized.

## Data Availability

Dataset available on request from the authors.
